# The Exposure Peaks of Traffic-Related Ultrafine Particles Associated with Inflammatory Biomarkers and Blood Lipid Profiles

**DOI:** 10.3390/toxics12020147

**Published:** 2024-02-13

**Authors:** Cheng Lin, Kevin J. Lane, Virginia R. Chomitz, Jeffrey K. Griffiths, Doug Brugge

**Affiliations:** 1 Department of Public Health and Community Medicine, Tufts University School of Medicine, Boston, MA 02111, USA; cheng.lin@tufts.edu (C.L.); virginia.chomitz@tufts.edu (V.R.C.); jeffrey.griffiths@tufts.edu (J.K.G.); 2 Department of Environmental Health, Boston University School of Public Health, Boston, MA 02118, USA; klane@bu.edu; 3 Department of Medicine, Tufts University School of Medicine and Tufts Medical Center, Boston, MA 02111, USA; 4 Department of Civil and Environmental Engineering, Tufts University School of Engineering, Medford, MA 02155, USA; 5 Department of Public Health Sciences, University of Connecticut School of Medicine, Farmington, CT 06030, USA

**Keywords:** ultrafine particle, UFP, peak exposure, inflammatory biomarker, blood lipid, exploratory research

## Abstract

In this article, we explored the effects of ultrafine particle (UFP) peak exposure on inflammatory biomarkers and blood lipids using two novel metrics—the intensity of peaks and the frequency of peaks. We used data previously collected by the Community Assessment of Freeway Exposure and Health project from participants in the Greater Boston Area. The UFP exposure data were time-activity-adjusted hourly average concentration, estimated using land use regression models based on mobile-monitored ambient concentrations. The outcome data included C-reactive protein, interleukin-6 (IL-6), tumor necrosis factor-alpha receptor 2 (TNF-RII), low-density lipoprotein (LDL), high-density lipoprotein (HDL), triglycerides and total cholesterol. For each health indicator, multivariate regression models were used to assess their associations with UFP peaks (N = 364–411). After adjusting for age, sex, body mass index, smoking status and education level, an increase in UFP peak exposure was significantly (*p* < 0.05) associated with an increase in TNF-RII and a decrease in HDL and triglycerides. Increases in UFP peaks were also significantly associated with increased IL-6 and decreased total cholesterol, while the same associations were not significant when annual average exposure was used. Our work suggests that analysis using peak exposure metrics could reveal more details about the effect of environmental exposures than the annual average metric.

## 1. Background and Introduction

Ultrafine particles (UFPs), usually referred to as airborne particles with a diameter of 100 nm or less (PM_0.1_), are a major component of particulate matter (PM) air pollutants [1]. In urban areas, motor vehicles are the leading source of UFP emissions [2,3,4], especially diesel and heavy-duty diesel vehicles whose exhaust consists of a large fraction of UFPs [2,5]. In total, gasoline and on-road diesel combustion contribute to about 50% of the UFP concentration in summertime in the eastern United States [6].

Traffic-related UFPs tend to concentrate in traffic corridors, areas in close proximity to major roadways. In locations beyond 100 m downwind of the road, the concentration of UFPs can drop by 50% [7,8]. However, many urban residents live closer than this. The US EPA estimated that 41 million, or 13% of the population of the contiguous US, live within 100 m of highways, arterial roads or other truck routes [9]. Another nationwide assessment revealed that between 2005 and 2006, 3.2 million (6.2%) American students in grades pre-K to 12 attended schools located within 100 m of a major roadway [10]. For these near-roadway populations, traffic-related UFPs have a disproportionate impact, justifying extra attention.

Due to their small size, UFPs can be deposited deep in the alveolar region of the lungs, where they can penetrate through the blood–gas barrier to enter the blood circulation. From there, they may further enter cells in various organs and interact directly with biomolecules [11,12]. UFPs can also be absorbed into nasal olfactory nerves and enter the central nervous system [12,13]. Recent toxicological studies have observed neurotoxicity effects associated with both short-term and long-term UFP exposure [14,15,16].

Moreover, UFPs have the potential to cause inflammatory reactions and oxidative stress in the human body [17,18], which can increase the risks for a variety of diseases, primarily those of the cardiovascular and respiratory systems [12,19,20,21]. It has been reported that UFPs are associated with increased levels of biomarkers of systemic inflammation [19,22,23,24]. There is also evidence that short- and long-term UFP exposure may be associated with elevated blood pressure as well as changes in blood lipid levels, especially a reduced high-density lipoprotein (HDL) level [25,26,27,28].

To our knowledge, studies about the health impact of UFPs focus on either long-term, population-based health indices associated with long-term exposure (e.g., annual average UFP concentration) [1,20,21] or short-term, acute health outcomes following short-term exposure events lasting for hours or days [23,29,30]. Here, we consider a third scenario, in which short-term high exposure peaks occur repeatedly and frequently over a relatively long period of time (e.g., a year). Our question is whether these exposure peaks might cumulatively contribute to long-term changes in health outcomes, as compared to a lower but steady level of exposure that persists over the same length of time.

We believe this question deserves further investigation because there is a possibility that repeated short-term peaks may overwhelm the human body’s physiological defensive responses or alter their protective mechanisms that could have otherwise mitigated UFP’s adverse effects [31]. Similar phenomena have been observed in studies of other environmental exposures such as ozone [32,33,34].

**Figure 1:**
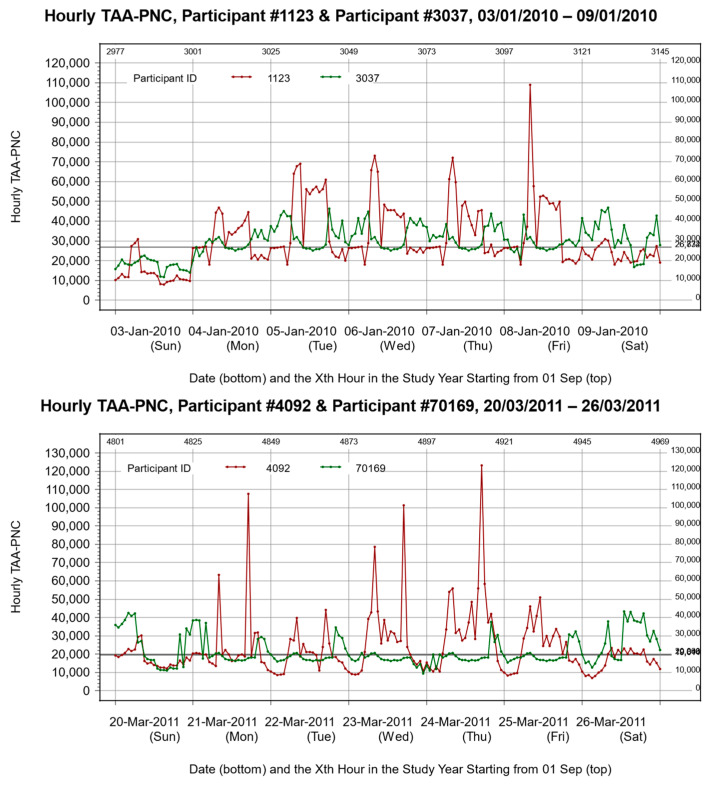
The fluctuation of in hourly TAA-PNC estimates during a one-week period for two pairs of participants. The pair of participants in the upper panel are from Somerville, and the pair of participants in the lower panel are from Dorchester–South Boston. In each panel, the two comparison participants are indicated by different colors (red and green), and the thicker horizontal line is actually two mostly-overlapping lines indicating annual averages of hourly TAA-PNC for the two participants, which are almost equal.

To facilitate our investigation, it was necessary to have a metric that could quantify the exposure from a series of peaks. The traditional average metrics cannot achieve this because they may not be able to distinguish exposure patterns with different numbers and shapes of peaks. For example, in Figure 1, each panel shows the exposure curves of two study participants whose annual average levels were almost indistinguishable despite having very different fluctuation patterns: one consisted of a distinct cluster of high peaks, while the other’s peaks were fewer in number and shorter.

In our previous work, we developed a set of two metrics measuring the overall magnitude of frequent and repeated exposure peaks cumulatively: (1) a metric reflecting the overall heights of the exposure peaks using a high percentile and (2) a metric reflecting the length of time (e.g., number of hours or days) during which the exposure level was above a threshold determined by a high percentile [35]. The concept of measuring peaks has been operationalized for other environmental exposures such as temperature (heat wave), which has a similar repetitive fluctuation pattern [36,37].

The Community Assessment of Freeway Exposure and Health (CAFEH) is a community-based research project aimed at assessing the health impacts of exposure to highway traffic-related air pollutants (especially UFP). The project used land-use regression (LUR) models and time-activity adjustment to predict UFP exposure for the study participants. It also collected a wide range of health data, including cardiovascular-disease-related inflammatory biomarkers and other cardiac health indices (such as blood lipids and blood pressure) [38,39,40]. Thus, the CAFEH project provides an appropriate data source for our study.

Prior analysis of CAFEH data involved only the inflammatory biomarker associations with traffic-related UFP exposure, as measured using annual averages [22]. An updated literature review indicated that UFP exposure might also be associated with changes in blood lipid levels [26,27,28]. Accordingly, in this study, in addition to introducing the new peak exposure metrics, we added blood lipid indicators as outcome variables.

## 2. Methods

Our study is a secondary analysis of data previously collected by the CAFEH project. Details of the data collection process have been published previously [22,38,39,40,41,42,43].

### 2.1. Study Area and Study Population

The CAFEH project was conducted in three separate study areas (namely, Somerville, Dorchester–South Boston and Chinatown/Malden) in the Greater Boston Area [43]. Each study area consisted of a pair of two sub-areas or neighborhoods: one near-highway neighborhood delimited within 500 m of the highway and one urban background neighborhood delimited 1 km or farther from the highway [22]. Both the monitoring of UFP concentrations and the recruitment of study participants took place in these areas.

The participants of the CAFEH studies were adults who were 40 years or older, living in the aforementioned study areas and able to complete health questionnaires [38]. They were recruited, and their data were collected, during the same one-year study period that the mobile-monitoring of UFP data (described below) was conducted in their study area. The one-year study periods were: from September 2009 to August 2010 in Somerville, from September 2010 to August 2011 in Dorchester–South Boston and from August 2011 to July 2012 in Chinatown/Malden [22,43]. Most of the participants were recruited using randomized sampling, while in each study area, a small convenience sample was also used. In the end, over 700 study participants were recruited [38,42].

### 2.2. Data Collection and Sample Size

Of the data collected by the CAFEH project, the following three types were used in our study:

1. Basic demographic and behavioral data.

These data were collected from the participants using self-reported questionnaires during in-home visits [38].

2. Outcome (health indicator) data.

The outcome variables included seven health indicators: three inflammatory biomarkers (C-reactive protein (CRP), interleukin-6 (IL-6) and tumor necrosis factor-alpha receptor 2 (TNF-RII)) and four blood lipid indicators (low-density lipoprotein (LDL), high-density lipoprotein (HDL), triglycerides (TG) and total cholesterol). They were all collected from the participants’ blood samples during a visit to a field clinic [38].

3. UFP exposure data.

These data were time-activity-adjusted hourly averages of UFP concentrations, which were estimated using land-use regression (LUR) models based on mobile-monitored ambient UFP concentrations. More details about the processing of the UFP data are described in Section 2.3.

We initially obtained records of 701 participants with complete UFP exposure estimates from CAFEH. Because the collection of blood samples was voluntary, not all participants completed blood tests. For each outcome (health indicator) variable, the number of participants with complete data of that variable ranged from 386 to 439. In total, 452 had complete data of at least one of the health indicators. After further excluding those without complete data of covariates, for each outcome variable, the final number of participants included in the analysis ranged from 364 to 411 (Table 1). 

### 2.3. Processing of UFP Exposure Data

As the first step, the raw data of the UFP concentration was collected through mobile-monitoring, using the Tufts Air Pollution Laboratory (TAPL). The TAPL was a vehicle with monitoring devices that could measure real-time air pollution levels while driving. Second-by-second data of the UFP concentration was measured as the number of particles per cubic centimeter (cm^3^) of ambient air, also known as the particle number concentration (PNC). The TAPL was driven repeatedly through local streets and highways along fixed paths in each of the three study areas during the corresponding one-year study period. To obtain a representative sample and to avoid autocorrelation during the modeling, the monitoring hours covered a majority of the hours in a day (from around 04:30 to 22:00) and were distributed evenly across all days of the week and all four seasons (21 to 70 h per season) in a year. It was determined that the number of monitoring hours was sufficient for characterizing PNC spatial variation [39,40,41].

Mobile-monitored UFP concentration (measured as PNC) data were then used to develop LUR models (based on log-linear regression) by adding the time, location, hourly traffic and meteorological variables specific to the neighborhoods as predictors. Four neighborhood-specific LUR models (corresponding to neighborhood(s) in Somerville, Dorchester–South Boston, Chinatown and Malden) were established. This allowed for an estimation of the hourly averaged UFP concentration for all locations in the study areas that were not on the TAPL path, with a resolution of about 20 m. All neighborhood-specific models were cross-validated. The performance of the models (measured by adjusted R^2^) was found to be comparable to that of similar models in other studies, while our models achieved a better temporal and spatial resolution [39,40]. Using the LUR models, for each participant, the hourly ambient UFP PNC level at their residence was estimated for every hour within the one-year study period. Because previous studies, both in the Chinatown neighborhood and in other areas, suggested that there was only little reduction of the UFP concentration with the vertical position [44,45], and because all recruited participants were living no higher than three floors above the street level, the vertical height of the participants’ homes was not considered an important factor when estimating the residential PNC.

The modeled PNC data were further modified by time-activity adjustment (TAA), to consider the time the participants spent in different micro-environments (in home (with or without air conditioning), at home outdoors, at work/school, on highways and in other environments) where the actual UFP exposure level might be different from the residential ambient exposure. TAA factored in this possibility and adjusted the exposure level accordingly. The assignment of micro-environments was based on the participants’ self-reported daily activities for each hour of the most recent workday and non-workday. The application of TAA reportedly reduced exposure misclassification [22,42].

### 2.4. Definition of Peak Exposure Metrics

**Figure 2:**
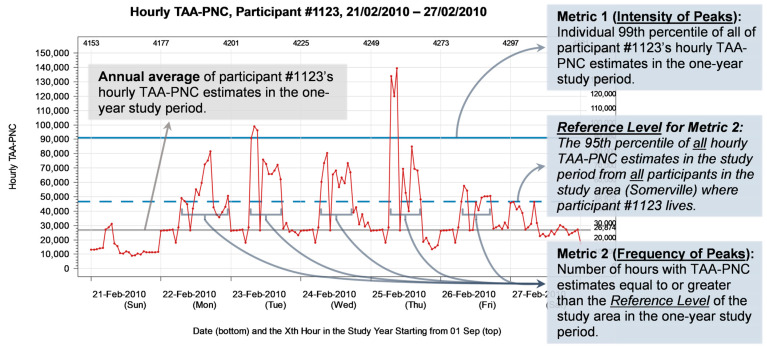
Definitions and levels of the annual average and peak exposure metrics (including the intensity and frequency of peaks), using participant #1123’s fluctuation of hourly TAA-PNC estimates by time during a week in February 2010 as an example. Note that our definitions do not mandate which cut-off percentile should be used. In this figure, for demonstration purposes, the cut-off percentile for the intensity metric was set at P99, and the cut-off percentile defining the reference level for the frequency metric was set at P95.

The time-activity-adjusted, LUR-model-estimated hourly average PNC (TAA-PNC) was the exposure data used for the annual average and peak exposure metrics in our analysis. More details about the development and characteristics of the peak exposure metrics have been described in our previous article [35]. Briefly, we introduced the following two peak exposure metrics (Figure 2):

1. The intensity of peaks.

The intensity of peaks was defined as a high percentile of exposure measurements of all time points within a given timeframe. In our study, specifically, a participant’s intensity of UFP exposure peaks was the high percentile of all their hourly TAA-PNC estimates over the one-year study period.

2. The frequency of peaks.

The frequency of peaks was defined as the length of time during which the exposure measurement exceeded a pre-set threshold within a given timeframe. In our study, specifically, the threshold, also known as the reference level of a study area, was defined as a high percentile of all hourly TAA-PNC estimates over the one-year study period from all study participants in the area, and a participant’s frequency of UFP exposure peaks was the number of hours within the study period during which the TAA-PNC estimate was equal to or greater than the reference level of their study area.

As this was an exploratory approach, for the aforementioned “high percentile”, we set four cut-off percentiles a priori as candidates: the 90th percentile (P90), the 95th percentile (P95), the 98th percentile (P98) and the 99th percentile (P99).

### 2.5. Statistical Analysis

We sought to make our results comparable to the previously published analysis, which used the same CAFEH data to test the associations between the inflammatory biomarkers and the UFP annual average [22]. For this purpose, we used similar generalized linear models that included the same covariates (age, gender, race/ethnicity, education level, smoking status and BMI). The only change was that we substituted the annual average exposure in the previous models with our peak exposure. We fit the models with each of the seven health indicators—three inflammatory biomarkers and four blood lipid indicators—as the primary outcome variable and each of the two UFP peak metrics as the primary exposure variable. We tested all four cut-off percentiles that were used to define the peak metrics.

For comparison purposes, using this same model, we also analyzed the associations of the seven health indicators with the annual average UFP exposure, including the associations of the three biomarkers that had been analyzed in the previous study [22].

In addition to the crude analysis, a stratified analysis based on race/ethnicity was also conducted, since the previous study suggested that the associations differed between non-Hispanic Whites and East Asians [22].

We examined the normality of all outcome variables (Figure S1, Supplemental Materials) before fitting them in the models and applied natural log transformation to five of them (the three inflammatory biomarkers plus TG and total cholesterol), because we considered them to be overly skewed away from normal distributions.

Based on the beta-coefficients (along with 95% confidence intervals) of the exposure variables in our models, we calculated and compared the effect size of the annual average and peak exposures. The effect size was the amount of change (for blood LDL and HDL levels) or the percentage of change (for blood CRP, IL-6, TNF-RII, TG and total cholesterol levels, which were log-transformed in the models) in the outcome variables relative to a 1000 cm^−3^ increase (for the TAA-PNC average and intensity of peaks) or a 50 h increase (for the TAA-PNC frequency of peaks) in the UFP exposure.

SAS 9.4 (SAS Institute Inc., Cary, CA, USA) was used to conduct all analyses and generate all figures and graphs in this article.

## 3. Results

### 3.1. Demographic Characteristics of the Study Population

Table 2 shows the demographic characteristics of the participants who were included in any of the regression models. A total of 422 participants had the complete data necessary for being included in the regression analyses. For most of the demographic factors, distributions were very similar between the near-highway and the urban background residents, except for the education level, which tended to be lower among participants living in near-highway areas. A majority of the study participants were either Non-Hispanic Whites or Asians, each of whom accounted for 40% of the total. Compared to the US nationwide population, the CAFEH study population over-represented Asians, enabling stratified analysis for the White and Asian populations. Stratified analysis for other races/ethnicities was not conducted due to the insufficient sample size.

### 3.2. Statistical Characteristics of UFP Exposure Metrics and Health Outcome Indicators

Table 3 lists descriptive statistics for the three UFP exposure measures—the two peak exposure metrics we developed and the traditional annual average exposure metric. Consistent with the previous study [22], the TAA-PNC annual average for near-highway participants was greater than for participants living farther from the highway (in urban background areas). This was also true when the intensity of peaks was used as the exposure metric. Similarly, when the frequency (duration) of peaks was used, near-highway participants had many more hours with TAA-PNC levels above the reference level. In brief, the peak and annual average exposure metrics agreed with each other in showing how the UFP exposure level differed between participants living in near-highway areas and those in the urban background areas.

We also examined the statistical distribution of the exposure metrics for each of the three study areas. While the exposure level varied across the three areas, the general pattern of the near-highway/urban-background difference was consistent, whether it was the annual average or the peak exposure metrics that were used (data not shown).

Table 4 compares the number of participants whose measures of the frequency of peaks were equal to zero, by different reference level cut-points (cut-off percentiles). When the reference level was set to the 98th or 99th percentile of all hourly TAA-PNC estimates from all participants in the study area, a significant portion (over 50% when the cut-off percentile was set at P99) of the participants living in urban background areas would not have any hour with a UFP concentration that exceeded the reference level. In contrast, many participants in near-highway areas were estimated to have over 100 h of peak-level UFP exposure. When the reference level was set lower (using P95 as the cut-point), there were far fewer (less than 5%) participants whose values of frequency of peaks were zero. 

Table 1 lists descriptive statistics for the seven outcome variables—the three inflammatory biomarkers and four blood lipid indicators. A small difference in the levels of many of the health indicators can be seen between participants living in near-highway areas and in urban background areas. For those in near-highway areas, blood CRP, IL-6, TG and total cholesterol tended to be slightly higher, while their blood HDL tended to be lower. However, these differences were not statistically significant. 

### 3.3. Health Indicators Associated with Both the UFP Annual Average and Peak Exposure

**Figure 3:**
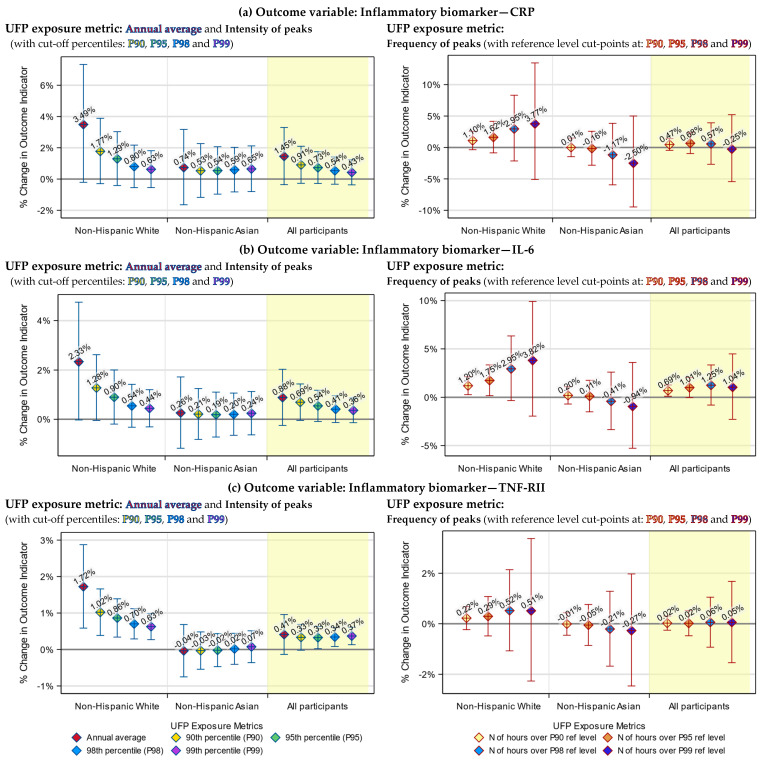
Model-estimated percentage of change in levels of inflammatory biomarkers, associated with a 1000 cm^−3^ increase in the annual average or intensity of peaks of ultrafine particles (UFP) exposure (left) or with a 50 h increase in the frequency of UFP exposure peaks (right), by cut-off percentiles and race strata. The blue vertical lines in the left panels indicate the 95% confidence intervals (95% CIs) of the estimated effects of the UFP annual average and intensity of peaks. The red vertical lines in the right panels indicate the 95% CIs of the estimated effects of the frequency of peaks.

**Figure 4:**
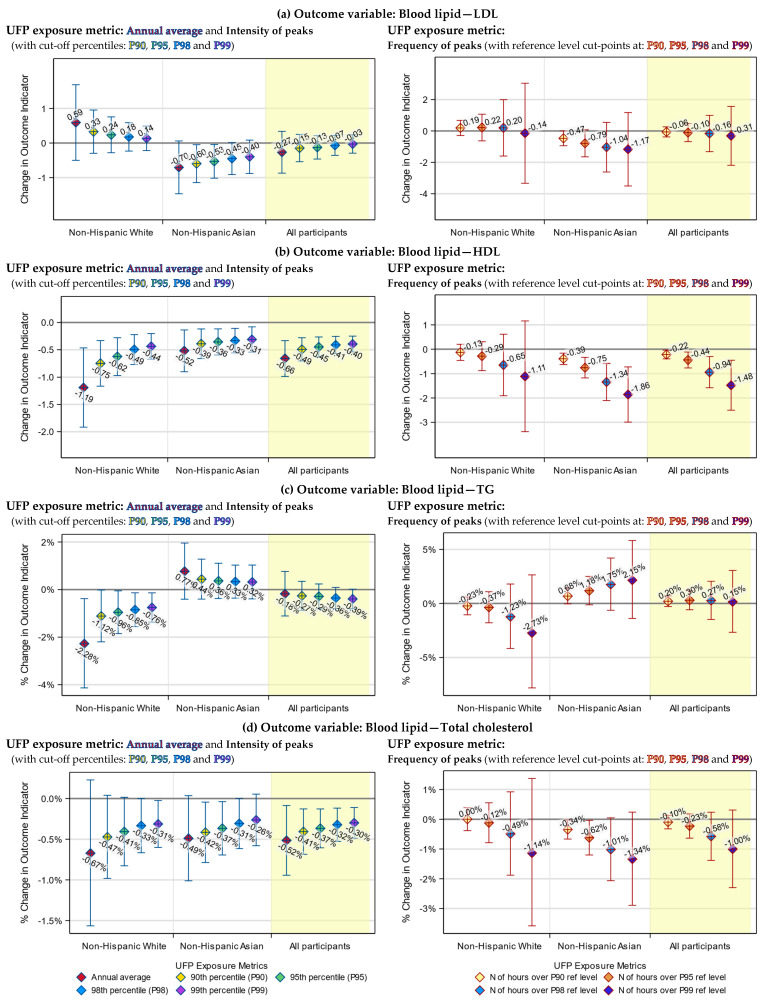
Model-estimated change (**a**,**b**) or percentage of change (**c**,**d**) in levels of blood lipid indicators, associated with a 1000 cm^−3^ increase in the annual average or intensity of peaks of UFP exposure (left) or with a 50 h increase in the frequency of UFP exposure peaks (right), by cut-off percentiles and race strata. The blue vertical lines in the left panels indicate the 95% CIs of the estimated effects of the UFP annual average and intensity of peaks. The red vertical lines in the right panels indicate the 95% CIs of the estimated effects of the frequency of peaks.

**Figure 5:**
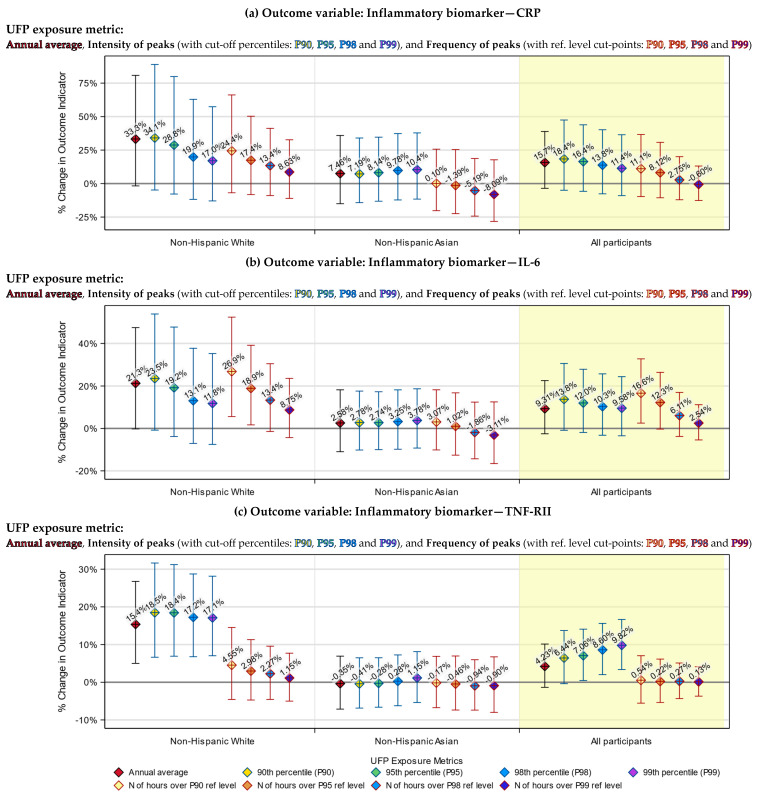
Model-estimated percentage of change in levels of inflammatory biomarkers, associated with one inter-quartile range’s (IQR) increase in the UFP annual average or peak exposure metrics, by cut-off percentiles and race strata. The black, blue and red vertical lines indicate the 95% CIs of the estimated effects of the UFP annual average exposure, intensity of exposure peaks and frequency of exposure peaks, respectively.

**Figure 6:**
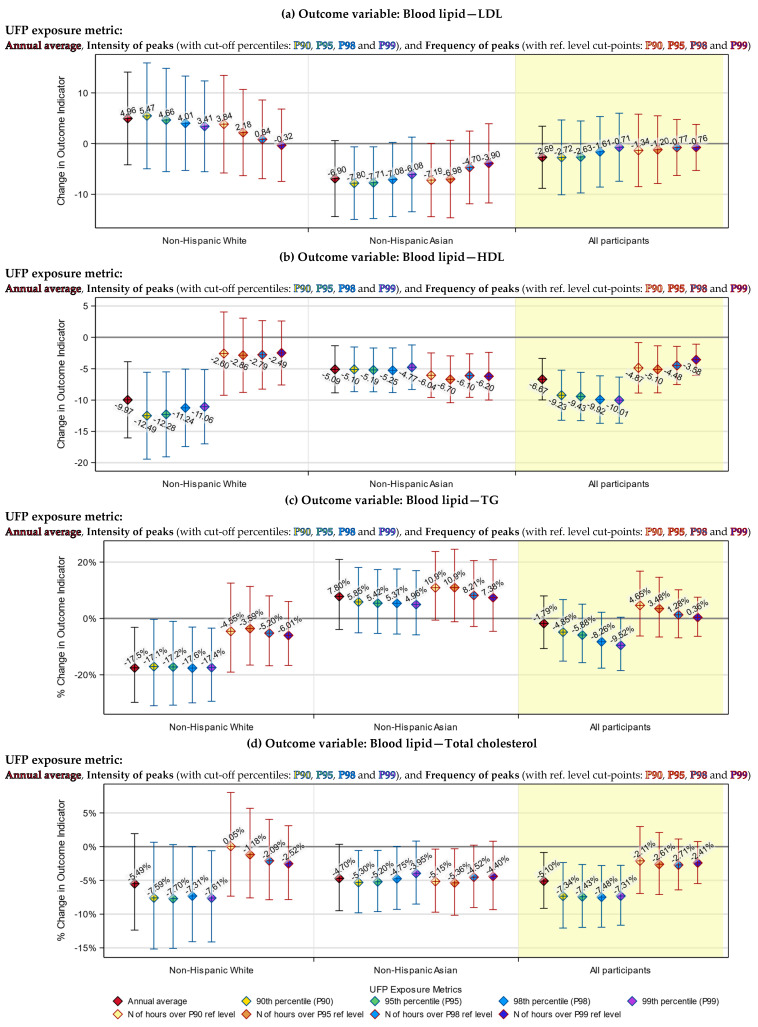
Model-estimated change (**a**,**b**) or percentage of change (**c**,**d**) in levels of blood lipid indicators, associated with one IQR’s increase in the UFP annual average or peak exposure metrics, by cut-off percentiles and race strata. The black, blue and red vertical lines indicate the 95% CIs of the estimated effects of the annual average exposure, intensity of exposure peaks and frequency of exposure peaks, respectively.

In our previous work, we determined that, for our dataset, P99 and P95 might be the best cut-off percentiles for defining the intensity and the frequency of UFP exposure peaks, respectively [35]. Therefore, although we tested all four percentiles, for simplicity purposes, only results based on these two percentiles are presented in Table 5. Results based on all four candidate percentiles are visualized in Figure 3, Figure 4, Figure 5 and Figure 6. 

We examined the association of UFP exposure with each of the seven health indicators, using both peak exposure (including intensity and frequency of peaks) and annual average exposure metrics. We found that the health indicators’ associations with the UFP peak exposure were generally consistent with the associations with the UFP annual average exposure.

Specifically, both the annual average and intensity of peaks of TAA-PNC were significantly associated with increases in the blood TNF-RII level among non-Hispanic Whites (*p* < 0.01), decreases in the blood TG level among non-Hispanic Whites (*p* < 0.05) and decreases in the blood HDL level among all participants (*p* < 0.0001).

The associations of these three health indicators with the frequency of peaks of TAA-PNC were mostly not statistically significant (*p* > 0.1, except for the association of HDL among Asians, which has a *p*-value < 0.001), yet they were in the same direction as the associations with the annual average (Table 5).

### 3.4. Health Indicators Associated with UFP Peak Exposure Only

There were a few associations that were only found using the peak exposure metrics. Among non-Hispanic Whites, the frequency of TAA-PNC peaks was significantly associated with an increase in the blood IL-6 level (*p* < 0.05). In comparison, only an insignificant association was found in the previous study when the annual average was used as the exposure metric [22]. Also, among the non-Hispanic Whites, the increase in the intensity of TAA-PNC peaks was significantly associated with a decrease in the blood total cholesterol level (*p* < 0.05), which was not found using the annual average (Table 5).

Likewise, among the Asian participants, increases in the frequency of TAA-PNC peaks were also significantly associated with a decreased level of total cholesterol (*p* < 0.05). In addition, for Asian participants, the intensity of TAA-PNC peaks was significantly associated with their LDL and total cholesterol level (*p* < 0.05), albeit only when P90 or P95 was used as the cut-off percentile. These three associations were not statistically significant when the annual average was used as the exposure metric (Table 5).

### 3.5. Racial Differences in the Responses to UFP Exposure

The associations of health indicators with TAA-PNC peak exposure had larger effect sizes (except the associations of LDL) and were more likely to be significant among non-Hispanic Whites than among East Asians. Particularly for TNF-RII and TG, significant associations were only found among the White participants (*p* < 0.001 for TNF-RII, and *p* < 0.05 for TG). Such a racial difference was also found in the associations with the annual average TAA-PNC.

For LDL and TG, there were divergent patterns of responses to UFP exposure between White and Asian participants, although the responses were not always statistically significant. Specifically, an increase in the annual average exposure or peak exposure of TAA-PNC tended to be associated with an increase in blood LDL and a decrease in TG among non-Hispanic Whites but with a decrease in LDL and an increase in TG among the Asians.

### 3.6. Differences between the Annual Average and Peak Exposure Metrics

Although the annual average exposure metric and our peak exposure metrics were broadly consistent in their associations with the health indicators, they still differed in ways that are worth being described. One of them is that the effect size of the model-estimated associations tended to be smaller for the intensity of exposure peaks than for the annual average. The other is that the effect size for the intensity of peaks had narrower 95% confidence interval (95% CI). Moreover, when the cut-off percentile of intensity of peaks was raised, we observed a trend by which the effect size got even smaller and the 95% CI became even narrower (Figure 3 and Figure 4). 

We were unable to directly compare the effect size for the frequency of exposure peaks to that for the annual average exposure since they used different units. Nevertheless, we saw that the trend of a changing effect size in response to a rising cut-off percentile was in the opposite direction compared to the same trend for the intensity of peaks: As the cut-point of the reference level was raised to a higher percentile, the effect size grew larger, while the 95% CIs became wider (Figure 3 and Figure 4). 

We also compared the changes in health indicators relative to one inter-quartile range’s (IQR) increase in the annual average exposure and peak exposure (Figure 5 and Figure 6). The changes relative to one IQR’s increase in the annual average and the changes relative to the same increase in peak exposure were generally comparable, except that, for outcome variables that were significantly associated with the annual average or intensity of peaks (TNF-RII, HDL, TG and total cholesterol), among the White participants, the changes relative to one IQR’s increase in the frequency of peaks were much smaller. The 95% CIs of the changes were also comparable, and when the cut-off percentile was raised, for both the intensity and frequency of peaks, the 95% CIs either remained unchanged or became somewhat narrower.

## 4. Discussion

### 4.1. Distribution Characteristics of UFP Peak Exposure

The statistical distribution of exposure metrics in Table 3 confirms that residents living near highways or other major roadways were exposed to higher UFP concentrations, regardless of which exposure metric was used. This tells us that participants living in near-highway areas experienced not only a higher UFP average exposure but also higher UFP exposure peaks than those living farther from the highway. Furthermore, the distribution of the frequency of exposure peaks shows that, although some participants living farther from the highway could be exposed to high peaks occasionally, the near-highway residents experienced these peaks far more often. This finding is consistent with traffic-related UFPs’ tendency of accumulating in narrow areas along traffic routes. It also reaffirms that our peak exposure metrics can reveal additional details about near-highway UFP exposure.

### 4.2. Associations between UFP Peak Exposure and Health Outcomes

We would like to emphasize that the peak exposure measured by our novel metrics was the overall or collective magnitude of all exposure peaks in a year, not the level of one or a few peaks, so the associations we found here should be seen as the associations of all exposure peaks over a year as a whole with the long-term health changes.

The associations we found between the UFP peak exposure and the inflammatory biomarkers were generally consistent with the results from the previous CAFEH study that used the annual average exposure metric [22]. This supports the idea that the peak exposure metrics could serve as supplementary, or even substitutable, metrics to the annual average.

Our tests of UFPs’ association with the blood lipid profiles were new for this dataset. The strongest association we found was a negative association between UFP exposure (both peak and annual average) and blood HDL. This finding is consistent with reports from previous studies on other populations [27,46,47]. More importantly, we also found that UFP peak exposure had significant associations with some other blood lipid indicators (e.g., total cholesterol), while the annual average exposure only had non-significant associations with these indicators. This again signifies the usefulness of the peak exposure metrics as a supplement to the traditional annual average exposure metric in uncovering associations that may otherwise be missed.

For certain health indicators such as CRP, LDL and TG (for Asian participants), even though their associations with UFP exposure were not significant, they tended to be in the same direction regardless of which exposure metric was used. That is, if a health indicator was positively associated with one exposure metric (for example, the UFP annual average), then its associations with all other UFP peak exposure metrics based on any of the four cut-off percentiles, whether significant or not, would also be positive, and vice versa. This implies that the relationships found with the annual average were robust to alternative exposure assessments, which justifies replacing the annual average exposure with peak exposure in the models. It also strengthens our confidence that those relationships might be more than a production of random effects and thus deserve further research and evaluation.

### 4.3. Differences between Racial Groups

When comparing the racial groups, we found different, sometimes even opposite, results between White and Asian participants. This happened not only to the UFPs’ associations with the inflammatory biomarkers, which had been reported in a previous study [22], but also to associations with the blood lipids that had not been analyzed before, implying that our Asian participants had a lower responsiveness to UFP exposure. Considering that the Asian participants in the CAFEH project were almost entirely foreign-born, it is possible their responsiveness to UFPs was related to the birthplace. This is consistent with the results of an earlier study that also took place in the Greater Boston area, in which birthplace was found to be a strong protective predictor of certain cardiopulmonary outcomes like asthma [48]. Meanwhile, it was noteworthy that, among the CAFEH participants, the first-generation Asian immigrants had lower inflammatory biomarker levels and healthier blood lipid profiles at the baseline [49]. Similar phenomena referred to as the “healthy immigrant effect” have been documented by other research as well [50,51].

### 4.4. Determination of Cut-off Percentiles for UFP Peak Exposure Metrics

Our methods for determining cut-off percentiles for the peak exposure metrics were exploratory. In our previous work, we observed that a higher cut-off percentile caused the UFP peak exposure measurement to be less correlated with the annual average, and we pointed out that this could make it more likely for the peak exposure metrics to find differences that had not been revealed by the annual average. However, for the frequency of peaks, when the cut-point of the reference level was set too high, it resulted in too many participants with a value of frequency of peaks equal to zero (Table 4), making the model estimates less accurate or meaningful [35].

In this study, we further observed that, when the intensity of peaks was used to measure the exposure, a higher cut-off percentile led to smaller effect sizes with narrower 95% CIs, whereas when the frequency of peaks was used, a higher cut-off percentile led to larger effect sizes with wider 95% CIs. The reduced effect size might be seen as hinting that the intensity of peaks is less sensitive for predicting the associations with health indicators. However, this was likely due to the fact that the intensity of peaks had a wider range of variation than the annual average (Table 3). As a result, for the same study sample, compared with the annual average exposure, the intensity of exposure peaks required a larger difference to be associated with the same amount of change in the health indicators. Similarly, for the frequency of peaks, when the cut-point of the reference level was raised, the range of variation became narrower, because the participants generally had fewer hours with UFP concentrations above the reference level. This explains why when comparing the changes in health indicators relative to one IQR’s increase, rather than to a fixed amount of increase, in the UFP annual average and peak exposure, the differences in effect sizes mostly diminished (Figure 5 and Figure 6).

Therefore, taking all of this into account, we continue to believe that the 99th percentile might be the best fit for the intensity of peaks, because it was correlated with the annual average the least and yielded similar or narrower 95% CIs, bringing forth a better chance of unveiling associations that were not seen by using the annual average. On the flip side, determining the optimal reference level cut-point for the frequency of peaks is more complicated. In both our previous work and this study, it appeared to us that the 95th percentile might achieve the best balance between reducing the correlation with the annual average and avoiding too many values of zero. Still, we refrain from saying with certainty that these cut-points are the best choice for every dataset. In fact, there might not be a universally applicable level where the cut-off percentiles should be set, especially for the frequency of exposure peaks. Our advice is that one should try several “candidate” cut-off percentiles and make adjustments based on preliminary findings.

### 4.5. Comparing UFP Peak Exposure Metrics

Comparing the two peak exposure metrics, the intensity of peaks appeared to be more useful as a supplemental exposure metric, especially among our non-Hispanic White participants, since it was more likely to have significant associations with the health indicators. For health indicators that had significant associations with the annual average, replacing the exposure metric with the intensity of peaks always yielded consistent results. On the other hand, the frequency of peaks appeared to perform better in our Asian sub-population, since there were more agreements between the two peak exposure metrics when the analysis was limited to Asians. At this point, we would advise against the use of frequency of peaks as the only exposure metric because, in our study, some of the significant associations found using the annual average or intensity of peaks could not be replicated using the frequency of peaks (e.g., the significant association with blood TNF-RII among non-Hispanic Whites).

Nevertheless, given the novelty of our approach and the fact that our analysis was limited to only one dataset, the results we have so far are still insufficient to support a confident decision about which peak exposure metric is generally better or which percentile threshold(s) should be chosen. Thus, we suggest applying both metrics in future research, as it is likely that different metrics in different study populations might show strengths in one case and not another.

### 4.6. Strengths and Limitations

It is worth reiterating that our study is exploratory, with the goal of applying novel metrics in a real-world epidemiological context to see how they perform against the traditional approach. For this reason, what we can provide at this stage is only a general concept or framework, rather than a completely established solution or guideline. More studies are needed to evaluate the use of the peak exposure metrics in analyses with different exposures, outcomes, populations, and study designs in order to find the best practices for using them in conjunction with other traditional metrics.

We were unable to fully disentangle the effect of the peak exposure from the effect of the annual average exposure. As an exploratory study, our analysis could not tell how much of the change in the inflammatory biomarkers or blood lipid indicators was due to the high peaks versus the average level of exposure. In part, this was because the peak exposure was still substantially correlated with the annual average in our UFP concentration data. As demonstrated in our previous work, depending on the different neighborhood-specific exposure patterns, the correlation coefficient of the relationship between the annual average and peak exposure ranged from 0.39 to 0.97 [35]. Consequently, there was strong multicollinearity between them, which prevented us from putting them in the same regression model to examine their effects independently. Nevertheless, this result told us that at least a part of the variation in the peak exposure is not attributable to the annual average exposure [35], which is why the peak exposure metrics are able to reveal more information about the exposure pattern and its associations with health indicators than the annual average. It would be interesting to see how our novel metrics work on other environmental exposures that can fluctuate extensively over time. We expect that the less the peak or extreme exposure levels are correlated with the long-term average, the more likely it is that our peak metrics would yield valuable findings.

One primary limitation of our study is related to the data we used. The biomarker and blood lipid data were cross-sectional and modest in terms of the sample size, which restricted us from drawing causal conclusions from the associations. Although cross-sectional data should be able to reflect reasonably well the participants’ long-term, chronic inflammatory and blood lipid condition, it was possible that some of the participants’ blood test results were influenced by acute health conditions or other factors unrelated to UFP exposure. A future longitudinal study collecting prospective health indicator data could better reflect the participants’ long-term health status and could address causality linkages if the health data were collected both before and after the exposure period of interest. As for the exposure data, only estimates of the UFP concentration were available for our analysis, so we had no chance to investigate potential interactions among different types of air pollutants. There were reports that traffic-related UFPs’ association with certain inflammatory biomarkers may be confounded by polycyclic aromatic hydrocarbons (PAHs) [52]. However, since our main goal was to test the novel peak exposure metrics using real-world data, this limitation did not affect our conclusion that peak exposure metrics are valid and can be a valuable supplement to, or substitute for, the average metrics.

Lastly, our UFP exposure data were estimated by LUR models rather than directly measured personal exposure data. Even though the application of the LUR model and the TAA approach has improved the accuracy and reduced the misclassification of exposure, it is likely the model estimates were still more or less different from the participants’ actual exposure levels. The estimates are good enough to produce statistics such as peak exposure metrics to quantify the overall magnitude of all the peaks as a whole, but they may be less useful if we want to precisely measure a short-term exposure event or one single peak in detail to evaluate its immediate health impact. Future researchers could consider using wearable monitoring devices to obtain UFP exposure data more accurately. Moreover, the LUR model was solely based on mobile-monitored data, which did not cover all 24 h of a day. A later study in the Boston area concluded that adding data from stationary monitoring sites could have improved the LUR model, as it better captured the nighttime reduction in PNC when the mobile-monitored data were scarce [53].

## 5. Conclusions

In this study, following up our previous work, we applied novel peak exposure metrics to real-world UFP concentration data for the first time and then examined their associations with inflammatory biomarkers and blood lipid indicators. The peak exposure metrics reflected the general exposure level of all the frequent and repeated peaks over the course of a year. We sought to find out whether these peaks, collectively, would have a long-term, cumulative impact on the health indicators.

We found the results were largely consistent, both externally across the three measures (annual average, intensity of peaks and frequency of peaks) and internally among the same peak exposure measure with different cut-off percentiles. We also found additional significant associations between the UFP peak exposure and some of the health indicators whose associations were not statistically significant when the annual average exposure metric was used.

Our study sheds light on the advantages and challenges of considering the cumulative peak exposure versus the annual average exposure alone. Our results suggest it might be of great value for peak exposure metrics to supplement or possibly substitute for the annual average exposure metric. Peak-exposure-based analyses may contribute to the understanding of how environmental exposures can drive health outcomes, which has not been clearly seen through analyses based on average exposure. This is especially relevant for environmental exposures like UFPs that fluctuate over wide ranges within a short distance and time period, resulting in numerous exposure peaks. We are hoping for more studies that apply these peak exposure metrics to a spectrum of environmental exposures in various research scenarios to further explore its full potential. 

## Figures and Tables

**Table 1:** Descriptive statistics of health outcomes, including three inflammatory biomarkers and four blood lipid indicators, by types of neighborhood areas.

Health Outcome Indicator	N *	Mean	Standard Deviation	Median	Minimum	Lower Quartile	Upper Quartile	Maximum
Inflammatory Biomarkers:								
CRP (mg/L)								
Near-highway areas	323	3.26	6.17	1.29	0.05	0.50	3.35	58.52
Urban background areas	88	2.52	4.25	1.11	0.06	0.51	2.33	30.99
All study areas	411	3.10	5.81	1.27	0.05	0.50	3.27	58.52
IL-6 (pg/mL)								
Near-highway areas	323	2.21	2.57	1.36	0.34	0.83	2.45	21.55
Urban background areas	88	1.55	1.46	1.15	0.31	0.75	1.75	8.77
All study areas	411	2.07	2.39	1.29	0.31	0.81	2.24	21.55
TNF-RII (pg/mL)								
Near-highway areas	323	2633.21	1394.61	2242.41	995.86	1812.80	2981.22	13,455.50
Urban background areas	88	2539.83	1151.53	2249.52	1091.80	1890.92	2797.49	9150.00
All study areas	411	2613.21	1345.49	2244.44	995.86	1838.81	2954.23	13,455.50
Blood Lipid Indicators:								
LDL (mg/dL)								
Near-highway areas	284	91.98	35.29	88.50	1.00	70.00	108.00	281.00
Urban background areas	80	93.45	29.14	88.00	38.00	72.00	106.50	196.00
All study areas	364	92.31	34.00	88.00	1.00	70.50	108.00	281.00
HDL (mg/dL)								
Near-highway areas	298	49.57	20.93	47.00	15.00	36.00	60.00	100.00
Urban background areas	85	52.48	20.93	51.00	15.00	39.00	67.00	100.00
All study areas	383	50.22	20.94	47.00	15.00	37.00	62.00	100.00
Triglycerides (mg/dL)								
Near-highway areas	318	129.95	86.12	110.50	11.00	63.00	166.00	500.00
Urban background areas	89	105.47	62.49	91.00	50.00	62.00	139.00	444.00
All study areas	407	124.59	82.10	100.00	11.00	63.00	160.00	500.00
Total cholesterol (mg/dL)								
Near-highway areas	319	167.86	48.50	165.00	100.00	134.00	193.00	400.00
Urban background areas	89	163.17	40.22	158.00	100.00	140.00	186.00	268.00
All study areas	408	166.84	46.81	162.50	100.00	135.00	190.00	400.00

* Number of participants with complete TAA-PNC exposure, covariates (age, sex, race/ethnicity, BMI, smoking status and education level) and corresponding health outcome data, which is the number of participants (sample size) included in the corresponding final models. Note that the sample size for the regression analysis for different health outcome indicators can be slightly different.

**Table 2:** Background and demographic characteristics of participants who were included in regression analysis, by types of neighborhood areas.

	Near-Highway Neighborhoods	Urban Background Neighborhoods	All Study Areas
	Mean (SD)	Mean (SD)	Mean (SD)
Age	61.01 (12.56)	60.75 (13.17)	60.95 (12.68)
Body Mass Index	27.64 (6.83)	27.24 (6.86)	27.55 (6.83)
	N (%)	N (%)	N (%)
Gender			
Female	193 (58.31%)	56 (61.54%)	249 (59.00%)
Male	138 (41.69%)	35 (38.46%)	173 (41.00%)
Race/Ethnicity			
Non-Hispanic White	138 (41.69%)	43 (47.25%)	181 (42.89%)
Non-Hispanic Black	23 (6.95%)	7 (7.69%)	30 (7.11%)
Non-Hispanic Asian	132 (39.88%)	36 (39.56%)	168 (39.81%)
Other	38 (11.48%)	5 (5.49%)	43 (10.19%)
Education level			
Lower than 12th Grade	125 (37.76%)	16 (17.58%)	141 (33.41%)
12th Grade (High school diploma)	100 (30.21%)	24 (26.37%)	124 (29.38%)
Some undergraduate college	74 (22.36%)	30 (32.97%)	104 (24.64%)
Some postgraduate	32 (9.67%)	21 (23.08%)	53 (12.56%)
Smoking status			
Non-smoker	157 (47.43%)	49 (53.85%)	206 (48.82%)
Former smoker	104 (31.42%)	24 (26.37%)	128 (30.33%)
Current smoker	70 (21.15%)	18 (19.78%)	88 (20.85%)
Employment status			
Not employed	205 (62.50%)	51 (57.30%)	256 (61.39%)
Employed or full-time student	123 (37.50%)	38 (42.70%)	161 (38.61%)
(Missing) ^2^	3	2	5
Birthplace			
Outside the United States	193 (58.48%)	41 (46.07%)	234 (55.85%)
In the United States	137 (41.52%)	48 (53.93%)	185 (44.15%)
(Missing) ^2^	1	2	3
Total ^1^	331	91	422

^1^ The total number of participants whose data were included in any of the final regression models. These participants should have complete data of the model covariates (age, gender, race/ethnicity, education level, smoking status and BMI), the UFP exposure estimates and any one of the seven outcome indicators. ^2^ Participants’ birthplace and employment status were not included in the final regression models, hence the small number of missing values.

**Table 3:** Descriptive statistics of UFP exposure ^1^, by exposure metrics and types of neighborhood areas.

	N ^2^	Mean	Standard Deviation	Median	Minimum	Lower Quartile	Upper Quartile	Maximum
Annual average of TAA-PNC exposure (unit: cm^−3^)								
Near-highway areas	513	2.33 × 10^4^	0.48 × 10^4^	2.35 × 10^4^	1.08 × 10^4^	2.02 × 10^4^	2.71 × 10^4^	3.47 × 10^4^
Urban background areas	188	1.30 × 10^4^	0.31 × 10^4^	1.20 × 10^4^	0.88 × 10^4^	1.03 × 10^4^	1.57 × 10^4^	2.37 × 10^4^
All study areas	701	2.06 × 10^4^	0.64 × 10^4^	2.14 × 10^4^	0.88 × 10^4^	1.54 × 10^4^	2.56 × 10^4^	3.47 × 10^4^
Intensity of TAA-PNC exposure peaks (unit: cm^−3^) ^3^								
Near-highway areas	513	6.09 × 10^4^	1.19 × 10^4^	6.39 × 10^4^	2.53 × 10^4^	5.48 × 10^4^	6.98 × 10^4^	8.19 × 10^4^
Urban background areas	188	3.82 × 10^4^	0.87 × 10^4^	3.96 × 10^4^	2.19 × 10^4^	3.16 × 10^4^	4.34 × 10^4^	9.12 × 10^4^
All study areas	701	5.48 × 10^4^	1.50 × 10^4^	5.90 × 10^4^	2.19 × 10^4^	4.21 × 10^4^	6.75 × 10^4^	9.12 × 10^4^
Frequency of TAA-PNC exposure peaks (unit: hours) ^4^								
Near-highway areas	513	577.39	337.73	526	1	392	735	2196
Urban background areas	188	42.59	77.64	27	0	12	58	958
All study areas	701	433.96	375.84	427	0	67	645	2196

^1^ Showing only statistics of peak exposure metrics based on the preferred cut-off percentiles (P99 for intensity of peaks and P95 for frequency of peaks). See Table S1 in Supplementary Materials for statistics of peak exposure metrics based on all four candidate percentiles. ^2^ Number of participants with complete TAA-PNC exposure data, of whom not all were included in the final models. ^3^ Using P99 as the cut-off percentile. ^4^ Using P95 as the cut-off percentile to determine the reference level.

**Table 4:** Number of participants with (N_1_) and without (N_2_) non-zero values of frequency of UFP exposure peaks, based on different cut-off percentiles (reference level cut-points), by types of neighborhood areas.

Cut-off Percentile ^1^	Near-Highway Neighborhoods				Urban Background Neighborhoods				All Study Areas		
	N_1_ ^2^ (%)	N_2_ ^3^ (%)	N_total_ ^4^		N_1_ ^2^ (%)	N_2_ ^3^ (%)	N_total_ ^4^		N_1_ ^2^ (%)	N_2_ ^3^ (%)	N_total_ ^4^
90th percentile	513 (100.00%)	0 (0.00%)	513		188 (100.00%)	0 (0.00%)	188		701 (100.00%)	0 (0.00%)	701
95th percentile	513 (100.00%)	0 (0.00%)	513		180 (95.74%)	8 (4.26%)	188		693 (98.86%)	8 (1.14%)	701
98th percentile	509 (99.22%)	4 (0.78%)	513		145 (77.13%)	43 (22.87%)	188		654 (93.30%)	47 (6.70%)	701
99th percentile	500 (97.47%)	13 (2.53%)	513		85 (45.21%)	103 (54.79%)	188		585 (83.45%)	116 (16.55%)	701

^1^ The percentile used to determine the overall reference level for the definition of the frequency of TAA-PNC exposure peaks. ^2^ Number of participants with a non-zero value of frequency of TAA-PNC exposure peaks. ^3^ Number of participants whose values of frequency of TAA-PNC exposure peaks were zero (participants who did not have any hour with a TAA-PNC estimate that reached the reference level). ^4^ Total number of participants with available data of frequency of TAA-PNC exposure peaks in the neighborhood areas.

**Table 5:** Results of the multilinear regression analysis, showing the model-estimated change in health indicators associated with a certain amount of increase in UFP exposure, by exposure metrics and racial groups.

Inflammatory Biomarker	%Change (95% CI)		
	Non-Hispanic White	Asian	All Participants
CRP			
Annual average exposure ^1^	3.49% (−0.21%, 7.33%) *	0.74% (−1.65%, 3.17%)	1.45% (−0.36%, 3.30%)
Intensity of exposure peaks ^2^	0.63% (−0.55%, 1.81%)	0.65% (−0.80%, 2.12%)	0.43% (−0.37%, 1.23%)
Frequency of exposure peaks ^3^	1.62% (−0.86%, 4.16%)	−0.16% (−2.82%, 2.58%)	0.68% (−0.97%, 2.35%)
IL-6			
Annual average exposure ^1^	2.33% (−0.03%, 4.75%) *	0.26% (−1.18%, 1.72%)	0.88% (−0.25%, 2.03%)
Intensity of exposure peaks ^2^	0.44% (−0.31%, 1.20%)	0.24% (−0.63%, 1.13%)	0.36% (−0.14%, 0.87%)
Frequency of exposure peaks ^3^	1.75% (0.16%, 3.36%) **	0.11% (−1.51%, 1.76%)	1.01% (−0.03%, 2.05%) *
TNF-RII			
Annual average exposure ^1^	1.72% (0.59%, 2.87%) ***	−0.04% (−0.75%, 0.68%)	0.41% (−0.13%, 0.96%)
Intensity of exposure peaks ^2^	0.63% (0.27%, 0.99%) ****	0.07% (−0.36%, 0.51%)	0.37% (0.13%, 0.61%) ***
Frequency of exposure peaks ^3^	0.29% (−0.48%, 1.08%)	−0.05% (−0.86%, 0.76%)	0.02% (−0.48%, 0.52%)
Blood Lipid Indicator	Change in mg/dL (95% CI)		
	Non-Hispanic White	Asian	All participants
LDL			
Annual average exposure ^1^	0.59 (−0.50, 1.68)	−0.70 (−1.47, 0.06) *	−0.27 (−0.87, 0.34)
Intensity of exposure peaks ^2^	0.14 (−0.22, 0.49)	−0.40 (−0.88, 0.08) ^#^	−0.03 (−0.29, 0.24)
Frequency of exposure peaks ^3^	0.22 (−0.63, 1.07)	−0.79 (−1.65, 0.07) *	−0.10 (−0.68, 0.47)
HDL			
Annual average exposure ^1^	−1.19 (−1.92, −0.47) ***	−0.52 (−0.90, −0.14) ***	−0.66 (−0.99, −0.33) ****
Intensity of exposure peaks ^2^	−0.44 (−0.67, −0.20) ****	−0.31 (−0.54, −0.08) ***	−0.40 (−0.54, −0.25) ****
Frequency of exposure peaks ^3^	−0.29 (−0.88, 0.31)	−0.75 (−1.17, −0.33) ****	−0.44 (−0.76, −0.12) ***
Blood Lipid Indicator	% Change (95% CI)		
	Non-Hispanic White	Asian	All participants
Triglycerides			
Annual average exposure ^1^	−2.28% (−4.13%, −0.38%) **	0.77% (−0.41%, 1.96%)	−0.18% (−1.11%, 0.76%)
Intensity of exposure peaks ^2^	−0.76% (−1.37%, −0.14%) **	0.32% (−0.39%, 1.03%)	−0.39% (−0.80%, 0.02%) *
Frequency of exposure peaks ^3^	−0.37% (−1.79%, 1.08%)	1.18% (−0.13%, 2.50%) *	0.30% (−0.59%, 1.19%)
Total cholesterol			
Annual average exposure ^1^	−0.67% (−1.56%, 0.23%)	−0.49% (−1.01%, 0.04%) *	−0.52% (−0.94%, −0.09%) **
Intensity of exposure peaks ^2^	−0.31% (−0.60%, −0.02%) **	−0.26% (−0.58%, 0.05%) ^#^	−0.30% (−0.49%, −0.11%) ***
Frequency of exposure peaks ^3^	−0.12% (−0.79%, 0.55%)	−0.62% (−1.20%, −0.04%) **	−0.23% (−0.63%, 0.18%)

* *p* < 0.1. ** *p* < 0.05. *** *p* < 0.01. **** *p* < 0.001. ^#^ *p* < 0.05 only when P90 or P95 was used as the cut-off percentile. ^1^ Showing the estimated change in health indicators per each 1000 cm^−3^’s increase in the annual average of TAA-PNC exposure. ^2^ Showing the estimated change in health indicators per each 1000 cm^−3^’s increase in the intensity of TAA-PNC exposure peaks (using P99 as the cut-off percentile). ^3^ Showing the estimated change in health indicators per each 50 h increase in the frequency of TAA-PNC exposure peaks (using P95 as the cut-off percentile for defining the reference level).

## Data Availability

The original contributions presented in the study were included in the article and supplementary materials. Further inquiries can be directed to the corresponding authors.

## Supplementary Materials

The following supporting information can be downloaded at: https://www.mdpi.com/article/10.3390/toxics12020147/s1. Figure S1: Histograms of all outcome variables; Table S1: Descriptive statistics of UFP average and peak exposure metrics, based on each of the four candidate percentiles, by type of neighborhood areas.
