# Supplementary material for: The Exposure Peaks of Traffic-Related Ultrafine Particles Associated with Inflammatory Biomarkers and Blood Lipid Profiles
Source: Toxics. 2024 Feb 13;12(2):147. doi: 10.3390/toxics12020147 (PMC10893127; doi:10.3390/toxics12020147)
Supplement: Supplementary file 1 [file toxics-12-00147-s001.zip › toxics-2746639-supplementary.pdf]

The Exposure Peaks of Traffic-Related Ultrafine Particles Associated with Inflammatory Biomarkers and Blood Lipid Profiles.

Cheng Lin, Kevin J. Lane, Virginia R. Chomitz, Jeffrey K. Griffiths and Doug Brugge

Supplemental Materials

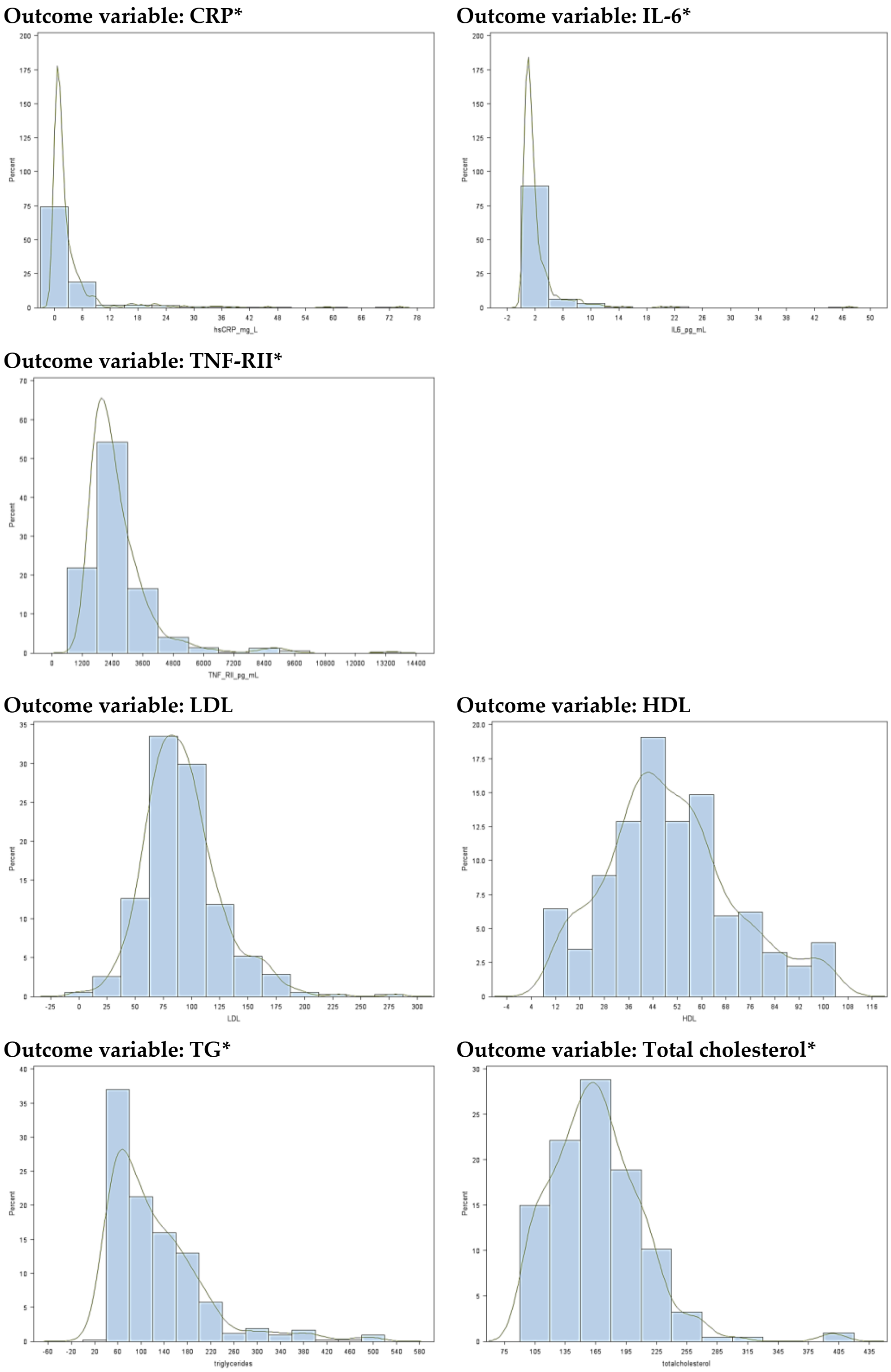

Figure S1. Histograms of all outcome variables. Out of the seven variables, five (CRP, IL-6, TNF-RII, TG and total cholesterol, marked by "*") were deemed overly skewed from a normal distribution, and were log-transformed before being added to the analysis.

Table S1. Descriptive statistics of UFP average and peak exposure metrics, based on each of the four candidate percentiles, by type of neighborhood areas.

	N [2]	Mean	Standard Deviation	Median	Minimum	Lower Quartile	Upper Quartile	Maximum
**Near-highway Neighborhoods**								
**Annual average of TAA-PNC exposure (Unit: cm^{-3})**								
	513	$2.33*10^{4}$	$0.48*10^{4}$	$2.35*10^{4}$	$1.08*10^{4}$	$2.02*10^{4}$	$2.71*10^{4}$	$3.47*10^{4}$
**Intensity of TAA-PNC exposure peaks (Unit: cm^{-3})**								
Cut-off percentile: P90	513	$3.86*10^{4}$	$0.82*10^{4}$	$4.05*10^{4}$	$1.73*10^{4}$	$3.48*10^{4}$	$4.42*10^{4}$	$5.50*10^{4}$
Cut-off percentile: P95	513	$4.58*10^{4}$	$0.96*10^{4}$	$4.83*10^{4}$	$1.97*10^{4}$	$4.14*10^{4}$	$5.21*10^{4}$	$6.39*10^{4}$
Cut-off percentile: P98	513	$5.49*10^{4}$	$1.11*10^{4}$	$5.77*10^{4}$	$2.21*10^{4}$	$4.87*10^{4}$	$6.25*10^{4}$	$7.27*10^{4}$
Cut-off percentile: P99	513	$6.09*10^{4}$	$1.19*10^{4}$	$6.39*10^{4}$	$2.53*10^{4}$	$5.48*10^{4}$	$6.98*10^{4}$	$8.19*10^{4}$
**Frequency of TAA-PNC exposure peaks (Unit: hours)**								
Ref. level cut-off pctl. [1]: P90	513	1138.24	557.05	1074	34	808	1462	3393
Ref. level cut-off pctl. [1]: P95	513	577.39	337.73	526	1	392	735	2196
Ref. level cut-off pctl. [1]: P98	513	234.03	178.07	199	0	123	293	1117
Ref. level cut-off pctl. [1]: P99	513	117.31	109.66	90	0	38	154	655
**Frequency of TAA-PNC exposure peaks, excluding zero values [3] (Unit: hours)**								
Ref. level cut-off pctl. [1]: P90	513	1138.24	557.05	1074	34	808	1462	3393
Ref. level cut-off pctl. [1]: P95	513	577.39	337.73	526	1	392	735	2196
Ref. level cut-off pctl. [1]: P98	509	235.86	177.55	201	1	123	294	1117
Ref. level cut-off pctl. [1]: P99	500	120.36	109.41	93.5	1	42.5	157.5	655
**Urban Background Neighborhoods**								
**Annual average of TAA-PNC exposure (Unit: cm^{-3})**								
	188	$1.30*10^{4}$	$0.31*10^{4}$	$1.20*10^{4}$	$0.88*10^{4}$	$1.03*10^{4}$	$1.57*10^{4}$	$2.37*10^{4}$
**Intensity of TAA-PNC exposure peaks (Unit: cm^{-3})**								
Cut-off percentile: P90	188	$2.27*10^{4}$	$0.36*10^{4}$	$2.17*10^{4}$	$1.68*10^{4}$	$2.03*10^{4}$	$2.50*10^{4}$	$4.81*10^{4}$
Cut-off percentile: P95	188	$2.70*10^{4}$	$0.48*10^{4}$	$2.69*10^{4}$	$1.83*10^{4}$	$2.41*10^{4}$	$3.02*10^{4}$	$5.91*10^{4}$
Cut-off percentile: P98	188	$3.34*10^{4}$	$0.71*10^{4}$	$3.44*10^{4}$	$2.07*10^{4}$	$2.85*10^{4}$	$3.73*10^{4}$	$7.61*10^{4}$
Cut-off percentile: P99	188	$3.82*10^{4}$	$0.87*10^{4}$	$3.96*10^{4}$	$2.19*10^{4}$	$3.16*10^{4}$	$4.34*10^{4}$	$9.12*10^{4}$
**Frequency of TAA-PNC exposure peaks (Unit: hours)**								
Ref. level cut-off pctl. [1]: P90	188	130.12	137.75	94	7	56.5	153.5	1391
Ref. level cut-off pctl. [1]: P95	188	42.59	77.64	27	0	12	58	958
Ref. level cut-off pctl. [1]: P98	188	8.63	36.25	2	0	1	6.5	471
Ref. level cut-off pctl. [1]: P99	188	3.52	21.26	0	0	0	1	277
**Frequency of TAA-PNC exposure peaks, excluding zero values [3] (Unit: hours)**								
Ref. level cut-off pctl. [1]: P90	188	130.12	137.75	94	7	56.5	153.5	1391
Ref. level cut-off pctl. [1]: P95	180	44.48	78.82	28	1	15	60.5	958
Ref. level cut-off pctl. [1]: P98	145	11.19	40.95	3	1	2	10	471
Ref. level cut-off pctl. [1]: P99	85	7.79	31.18	1	1	1	5	277
**All study areas**								
**Annual average of TAA-PNC exposure (Unit: cm^{-3})**								
	701	$2.06*10^{4}$	$0.64*10^{4}$	$2.14*10^{4}$	$0.88*10^{4}$	$1.54*10^{4}$	$2.56*10^{4}$	$3.47*10^{4}$
**Intensity of TAA-PNC exposure peaks (Unit: cm^{-3})**								
Cut-off percentile: P90	701	$3.44*10^{4}$	$1.01*10^{4}$	$3.69*10^{4}$	$1.68*10^{4}$	$2.45*10^{4}$	$4.32*10^{4}$	$5.50*10^{4}$
Cut-off percentile: P95	701	$4.07*10^{4}$	$1.19*10^{4}$	$4.37*10^{4}$	$1.83*10^{4}$	$2.94*10^{4}$	$5.04*10^{4}$	$6.39*10^{4}$
Cut-off percentile: P98	701	$4.91*10^{4}$	$1.39*10^{4}$	$5.27*10^{4}$	$2.07*10^{4}$	$3.66*10^{4}$	$6.07*10^{4}$	$7.61*10^{4}$
Cut-off percentile: P99	701	$5.48*10^{4}$	$1.50*10^{4}$	$5.90*10^{4}$	$2.19*10^{4}$	$4.21*10^{4}$	$6.75*10^{4}$	$9.12*10^{4}$
**Frequency of TAA-PNC exposure peaks (Unit: hours)**								
Ref. level cut-off pctl. [1]: P90	701	867.88	657.10	889	7	178	1291	3393
Ref. level cut-off pctl. [1]: P95	701	433.96	375.84	427	0	67	645	2196

Ref. level cut-off pctl. [1]: P98	701	173.58	183.11	143	0	11	249	1117
Ref. level cut-off pctl. [1]: P99	701	86.79	107.06	56	0	2	123	655
**Frequency of TAA-PNC exposure peaks, excluding zero values [3] (Unit: hours)**								
Ref. level cut-off pctl. [1]: P90	701	867.88	657.10	889	7	178	1291	3393
Ref. level cut-off pctl. [1]: P95	693	438.97	375.08	431	1	71	647	2196
Ref. level cut-off pctl. [1]: P98	654	186.05	183.35	156	1	24	260	1117
Ref. level cut-off pctl. [1]: P99	585	104.00	109.29	78	1	18	143	655

[1] The percentile used to determine the overall reference level for the definition of frequency of TAA-PNC exposure peaks.
[2] Number of participants with complete TAA-PNC exposure data, of whom not all were included in the final models.
[3] Including only participants with non-zero values of frequency of TAA-PNC exposure peaks.
